# Staging the Tumor and Staging the Host: Pretreatment Combined Neutrophil Lymphocyte Ratio and Modified Glasgow Prognostic Score Is Associated with Overall Survival in Patients with Esophagogastric Cancers Undergoing Treatment with Curative Intent

**DOI:** 10.1245/s10434-020-09074-5

**Published:** 2020-09-05

**Authors:** Stephen T. McSorley, Hiu Y. N. Lau, David McIntosh, Matthew J. Forshaw, Donald C. McMillan, Andrew B. Crumley

**Affiliations:** 1 Academic Unit of Surgery, New Lister Building, Glasgow Royal Infirmary, University of Glasgow, Glasgow, UK; 2 Beatson West of Scotland Cancer Centre, Glasgow, UK; 3 Department of Upper GI Surgery, Queen Elizabeth Building, Glasgow Royal Infirmary, Glasgow, UK; 4 Forth Valley Royal Hospital, Larbert, UK

## Abstract

**Background:**

This study examined whether an innate systemic inflammatory response (SIR) measured by combination neutrophil to lymphocyte ratio (NLR) and modified Glasgow Prognostic Score (mGPS) was associated with overall survival (OS) in patients with esophagogastric cancer (EC) undergoing neoadjuvant chemotherapy (NAC) followed by surgery.

**Methods:**

Patients diagnosed with EC, managed with NAC prior to surgery at a regional referral center, between January 2010 and December 2015, were included. The mGPS and NLR were calculated within 12 weeks before NAC. Patients were grouped by combined NLR/mGPS score into three groups of increasing SIR: NLR ≤ 3 (*n* = 152), NLR > 3 + mGPS = 0 (*n* = 55), and NLR > 3 + mGPS > 0 (*n* = 32). Univariable and multivariable Cox regression was used to analyse OS.

**Results:**

Overall, 337 NAC patients were included, with 301 (89%) proceeding to surgery and 215 (64%) having R0 resection. There were 203 deaths, with a median follow-up of those alive at censor of 69 months (range 44–114). Higher combined NLR/mGPS score (*n* = 239) was associated with poorer OS independent of clinical stage and performance status (hazard ratio 1.28, 95% confidence interval 1.02–1.61; *p* = 0.032), higher rate of progression on NAC (7% vs. 7% vs. 19%; *p* = 0.003), and lower proportion of eventual resection (80% vs. 84% vs. 53%; *p* = 0.003).

**Conclusions:**

The combined NLR/mGPS score was associated with OS and initial treatment outcomes in patients undergoing NAC prior to surgery for EC, stratifying survival in addition to clinical staging and performance status. The host SIR may be a useful adjunct to multidisciplinary decision making.

**Electronic supplementary material:**

The online version of this article (10.1245/s10434-020-09074-5) contains supplementary material, which is available to authorized users.

Esophagogastric cancers (ECs) are associated with poor survival,^1^ varying between 40% and 50% at 3 years for squamous cell carcinoma and adenocarcinoma, respectively, when treated with curative intent.^2^ Many patients present with advanced disease at diagnosis, decreasing the overall 5-year survival from 39% for localized disease to 4% for disease with distant metastases.^3^ Currently, patients with operable tumors are offered surgery with curative intent, often preceded by neoadjuvant chemotherapy (NAC). These patients undergo rigorous anesthetic assessment and cardiopulmonary exercise testing (CPET) prior to surgery. High rates of postoperative morbidity and mortality remain.^4^–^6^

Unfortunately, tumor recurrence is common even after treatment with curative intent, and the development of recurrent disease typically occurs within 2 years of surgery in 50% of resected patients.^7^ The main determinants of prognosis are pathological characteristics of the resected tumor specimen, including TNM stage, tumor differentiation, lymphovascular invasion (LVI), and resection margin involvement^8^,^9^; however, pretreatment clinical staging is also of prognostic importance.

In recent years, the presence of a preoperative innate systemic inflammatory response (SIR) has also been recognized as a potentially important prognostic marker in EC, in both palliative and curative settings.^10^ It is postulated to be related to the upregulation or inappropriate activation of the innate immune response, which suppresses the more useful anticancer adaptive response.^11^ It is therefore speculated that the presence of an innate SIR, represented by measurements of the acute-phase reactants C-reactive protein (CRP) and albumin in the form of the modified Glasgow Prognostic Score (mGPS) may be useful to identify EC patients who will have higher rates of postoperative morbidity and poorer prognosis following resection of locally advanced disease.^12^ Another commonly used measure of the SIR is the neutrophil to lymphocyte ratio (NLR).^13^ Recently, it has been proposed that mGPS and NLR be used together in a combined prognostic SIR score, providing gross and clinically available information relating to cytokine production and protein turnover, along with leukocyte and marrow responses.^14^

This study aimed to assess the combined NLR/mGPS score method prior to commencing NAC for EC patients treated with curative intent. Prognostic stratification with such a score, especially used together with clinical TNM staging, may be of value to prevent individuals with a particularly poor prognosis from undergoing chemotherapy, and then major surgery, resulting in significant morbidity, while also identifying those who would benefit from an aggressive approach.

## Patients and Methods

### Patients

This study included patients with EC treated with initial curative intent in the National Health Service (NHS) Greater Glasgow and Clyde (NHSGGC) and NHS Forth Valley (NHSFV) areas between January 2010 and December 2015. Patients not included were those who underwent potentially curative surgery without NAC, radical chemoradiation without any plan for surgery, those diagnosed with metastatic disease, and those who received palliative first-line treatment such as palliative chemotherapy and/or radiotherapy and any other palliative intervention, including laser, stent and dilatation.

All patients were discussed at a specialist multidisciplinary esophagogastric meeting following diagnosis, prior to NAC, prior to surgery, and then following surgery. NAC was offered to patients with an Eastern Cooperative Oncology Group (ECOG) score of 0–2 with clinical T3-4 disease or any clinical node-positive disease, or where there were concerns regarding possible margin-threatening disease at staging.

During staging, all patients underwent diagnostic upper gastrointestinal endoscopy followed by contrast computed tomography (CT) of the thorax, abdomen and pelvis. Positron emission tomography (PET)/CT was used in the majority of esophageal (94%) and junctional tumors (67%), and in a small proportion of gastric tumors (21%). Diagnostic laparoscopy, including washings-based cytology, was undertaken in 77% of esophageal tumors and 86% of junctional and gastric tumors. Endoscopic ultrasound was not used routinely but was used in a focused manner in situations of diagnostic or luminal staging doubt. No included patients underwent esophageal stenting, laser, or dilatation prior to treatment; however, a fine-bore enteral feeding tube was placed endoscopically in some cases of near-obstructing tumor.

All patients received NAC in either NHSGGC or NHSFV, and those who proceeded to surgery were operated on in a single tertiary referral teaching hospital (Glasgow Royal Infirmary). All NAC regimens used included a combination of a platinum-based drug plus 5-fluorouracil or capecitabine, as well as epirubicin where tolerated. There was a median of 8 weeks between the end of treatment and surgery, during which restaging occurred.

Patients with EC underwent Ivor Lewis, left thoracoabdominal, three-stage, or transhiatal esophagectomy, dependent on tumor site and surgeon preference. Patients with gastric cancer received either partial or total gastrectomy. At the induction of anesthesia, prophylactic antibiotics were administered. Venous thromboprophylaxis, in the form of pneumatic compression stockings and subcutaneous low molecular weight heparin, was given as per unit policy. During the initial postoperative recovery period, all patients were admitted to either the intensive care unit or surgical high dependency unit. Those patients who made sufficient progress were then transferred to the surgical ward. Patients were kept nil by mouth until the integrity of the anastomosis was confirmed using a water-soluble contrast swallow test, typically between postoperative days 6 and 9. During this period, nutrition was given either parenterally via a tunneled central venous catheter or via a feeding jejunostomy. Patients were reviewed by the surgical team daily and had daily postoperative blood tests, including full blood count (FBC), CRP, and albumin. Investigation and management of possible postoperative complications was at the discretion of the clinical team. This study was approved by the local Caldicott Guardian and Research Ethics Committee (19/SC/0653).

## Methods

The dataset was obtained from two prospectively maintained clinical databases: the Beatson Oncology Centre Chemocare database and the esophagogastric unit’s clinical outcomes audit database, which were used to identify patents who had NAC and surgery, respectively. The Community Health Index (CHI) number was used as the linkage variable to merge the data. Clinicopathological data were collected from electronic case notes, including age, sex, body mass index (BMI), smoking, tumor site, clinical and pathological TNM staging, preoperative investigations, hematological results, and inflammatory markers.

Pretreatment physical fitness was determined primarily based on performance status according to the ECOG score, oxygen uptake at the aerobic threshold (VO_2_ AT), and maximal oxygen uptake (VO_2_ peak) measured by CPET. CPET served as a semiquantitative functional assessment of cardiopulmonary reserve and can be used to predict the risk of perioperative morbidity and mortality. Thresholds of VO_2_ AT < 11 mL/kg/min and VO_2_ peak < 19 mL/kg/min were set, values below which were considered as having a certain level of cardiopulmonary dysfunction.^15^

The mGPS and NLR were used to quantify the SIR of the patients. The mGPS was calculated for each patient using serum CRP and albumin levels obtained from biochemistry reports. CRP and albumin were measured using an Abbot Architect (Abbot UK) multianalyzer. The scoring system for mGPS is as follows: CRP ≤ 10 mg/L = 0; CRP > 10 mg/L and albumin ≥ 35 g/L = 1; and CRP > 10 mg/L and albumin < 35 g/L = 2.^12^ A higher score reflects a more profound SIR. NLR was calculated by the number of neutrophils divided by the number of lymphocytes using values obtained from FBCs. A threshold of NLR > 3 was used to indicate a significantly elevated SIR.^13^ Prechemotherapy mGPS and NLR were calculated using reports at up to 3 months prior to chemotherapy and before any intervention such as diagnostic laparoscopy, or at the commencement of chemotherapy. Patients were grouped by combined NLR and mGPS into three groups, from least to most inflamed: NLR ≤ 3, NLR > 3 + mGPS = 0, NLR > 3 + mGPS > 0.

Patient’s staging CT scans were used to obtain the appropriate body composition measurement using ‘ImageJ’ (version 1.51, NIH, USA), a freeware validated software program. Axial CT slices were obtained at the level of the third lumbar vertebra. Region of interest (ROI) area measurements were skeletal muscle area (SMA) expressed as cm^2^ using standard Hounsfield unit (HU) ranges: − 29 to + 150 HU. SMA was then normalized to height (m) squared to create the skeletal muscle index (SMI), expressed as cm^2^/m^2^. Sarcopenia was defined as SMI < 52.3 cm^2^/m^2^ if the BMI was < 30 kg/m^2^, or SMI < 54.3 cm^2^/m^2^ if the BMI was ≥ 30 kg/m^2^ in male patients; and SMI < 38.6 cm^2^/m^2^ if the BMI was < 30 kg/m^2^, or SMI < 46.6 cm^2^/m^2^ if the BMI was ≥ 30 kg/m^2^ in female patients.^16^

### Statistical Analysis

Data analysis was performed in an anonymized manner using IBM SPSS software version 24 (SPSS, Inc., Chicago, IL, USA). The Pearson Chi square test was used to examine the associations between categorical variables, and the Chi square test for linear trend was used for ordered variables with multiple categories. Cox regression was used for univariable and then multivariable survival analysis for those variables found to be statistically significantly associated with overall survival (OS) at univariable analysis. All regressions were performed using a backward conditional model. Kaplan–Meier analysis and the log-rank test were used to examine OS pooled across clinical stage and performance status subgroups. OS was defined as the time from the date of diagnosis to the date of death due to any cause. A *p* value < 0.05 (two-sided) was considered statistically significant in all tests.

## Results

### Patients

The study included 337 patients (Table 1), with the majority being male (*n* = 240, 71%) and over 65 years of age (*n* = 175, 54%); 311 had adenocarcinoma at biopsy (93%), with 22 having squamous cell carcinoma (7%). There were 94 esophageal cancers (29%), 205 junctional cancers (62%), and 29 gastric cancers (9%). The majority had clinical T3-4 disease (*n* = 215, 65%) and clinical node-positive disease (*n* = 193, 59%) at pretreatment staging. The majority were reported to be ECOG performance status 0 or 1 prior to commencing NAC (*n* = 313, 96%).

**Table 1:** Clinicopathological characteristics among esophagogastric cancer patients prior to neoadjuvant chemotherapy with curative intent

Characteristic		All [*n* (%)]
*N*		337 (100)
*Patient characteristics*		
Age, years	< 65	149 (46)
	65–75	151 (47)
	> 75	24 (7)
Sex	Male	240 (71)
	Female	97 (29)
BMI, kg/m^2^	< 20	16 (5)
	20–24	103 (33)
	25–29	127 (40)
	> 29	69 (22)
Smoking	Never	109 (34)
	Ex-smoker	148 (46)
	Current	63 (20)
ECOG	0	234 (72)
	1	79 (24)
	2	14 (4)
VO_2_ AT, mL/kg/min [*n* = 255]	< 11	96 (38)
	≥ 11	159 (62)
VO_2_ peak, mL/kg/min [*n* = 204]	< 19	106 (52)
	≥ 19	98 (48)
Anemia [*n* = 295]	None	196 (66)
	Microcytic	19 (6)
	Normocytic	76 (26)
	Macrocytic	4 (2)
CT sarcopenia^a^	No	107 (36)
	Yes	187 (64)
*Tumor characteristics*		
Tumor site	Esophageal	94 (29)
	Gastric	29 (9)
	Junctional	205 (62)
Histology	Adenocarcinoma	311 (93)
	Squamous cell carcinoma	22 (7)
cTNM stage	1	32 (10)
	2	78 (23)
	3	208 (63)
	4A	14 (4)
*Systemic inflammation*		
mGPS [*n* = 264]	0	194 (73)
	1	41 (16)
	2	29 (11)
NLR [*n* = 293]	< 3	183 (54)
	≥ 3	110 (32)
*Initial treatment outcome*		
Surgery	Yes—resected R0	215 (64)
	Yes—resected R1/R2	55 (16)
	Yes—inoperable	31 (9)
	No—progressed on NAC	26 (8)
	No—medical complication on NAC	10 (3)

*BMI* body mass index, *ECOG* Eastern Cooperative Oncology Group performance status, *VO2 AT* oxygen uptake at the aerobic threshold, *VO2 peak* maximal oxygen uptake, *cTNM stage* clinical TNM stage, *mGPS* preoperative modified Glasgow Prognostic score, *NLR* neutrophil to lymphocyte ratio, *NAC* neoadjuvant chemotherapy, *CT* computed tomography, *SMI* skeletal muscle index

^a^CT sarcopenia was defined as SMI^b^ < 52.3 cm^2^/m^2^ if the BMI was < 30 kg/m^2^, or < 54.3 cm^2^/m^2^ if the BMI was ≥ 30 kg/m^2^ in men; and SMI < 38.6 cm^2^/m^2^ if the BMI was < 30 kg/m^2^, or < 46.6 cm^2^/m^2^ if the BMI was ≥ 30 kg/m^2^ in women

^b^SMI (cm^2^/m^2^) was defined as the skeletal muscle area (cm^2^) measured from the axial CT slice at the L3 vertebral level/height (m)^2^

All 337 patients received NAC, of whom 298 (88%) had one of the following regimens: epirubicin, cisplatin/carboplatin/oxaliplatin, 5-fluorouracil, capecitabine (ECF/ECX/EOF) and 39 patients (12%) had cisplatin and 5-fluorouracil/capecitabine. The median time between the completion of NAC and surgery was 8 weeks (interquartile range 7–9).

**Fig. 1:**
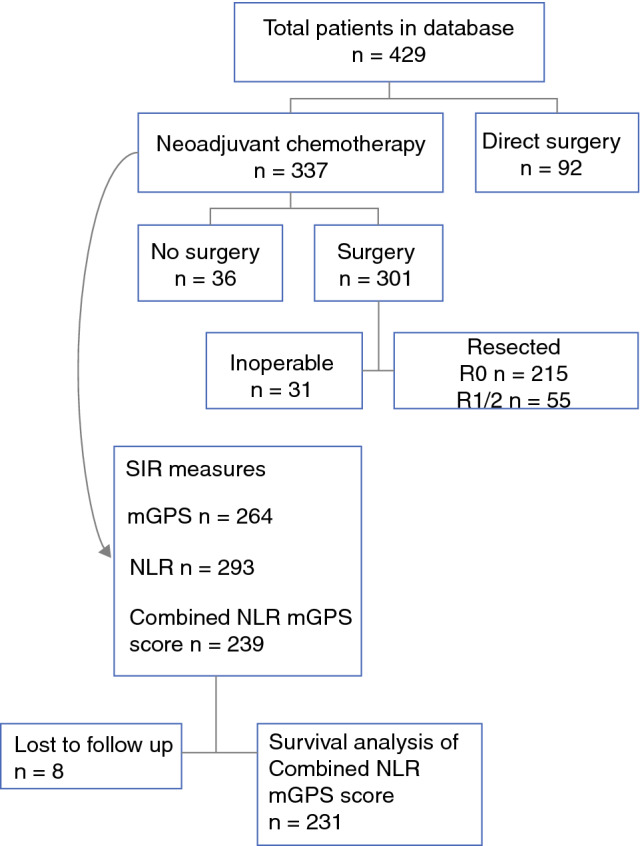
Patient inclusion process. *SIR* systemic inflammatory response, *mGPS* preoperative modified Glasgow Prognostic score, *NLR* neutrophil to lymphocyte ratio

Of the 337 patients, 215 (64%) had an R0 resection, 55 (16%) had an R1 or R2 resection, 31 (9%) had trial dissection but were found to be irresectable, 26 (8%) did not proceed to surgery due to disease progression during NAC, and 10 (3%) did not proceed to surgery due to a significant medical complication or toxicity during NAC (Fig. 1).

During the follow-up period there were 203 deaths (60%), with 183 of these deaths caused by EC. The median survival of the cohort as a whole was 37 months (interquartile range 25–48). The median follow-up of those alive at the time of censoring was 69 months (range 44–114).

### Creation of Combined Neutrophil to Lymphocyte Ratio (NLR)/Modified Glasgow Prognostic Score (mGPS) Score

Pretreatment mGPS could be calculated for 264 patients, NLR could be calculated for 293 patients, and was available for both in 239 patients, with survival data for 231 patients. Combining mGPS and NLR (Table 2) significantly stratified the 5-year percentage of OS more effectively than either measure of the SIR alone (*p* = 0.012). The data suggested that those with an NLR ≤ 3 have a similar 5-year percentage of OS regardless of the mGPS (0 = 47%, 1 = 55%, 2 = 45%), whereas those with an NLR > 3 had a poorer 5-year percentage of OS with increasing mGPS (0 = 34%, 1 = 29%, 2 = 21%). Therefore, a combined NLR/mGPS score was created for three groups: NLR ≤ 3 (*n* = 152), NLR > 3 + mGPS = 0 (*n* = 55), and NLR > 3 + mGPS > 0 (*n* = 32).

**Table 2:** Systemic inflammatory response measures associated with overall survival in esophagogastric cancer patients prior to neoadjuvant chemotherapy with initial curative intent

Proportion of patients surviving 5 years after diagnosis					
mGPS	NLR < 3		NLR ≥ 3		*p* Value
	*n*	5-year % OS (SE)	*n*	5-year % OS (SE)	
0	116	47 (5)	55	34 (6)	
1	20	55 (11)	17	29 (11)	
2	11	45 (15)	14	21 (11)	
					0.012

*mGPS* preoperative modified Glasgow Prognostic score, *NLR* neutrophil to lymphocyte ratio, *OS* overall survival, *SE* standard error

### Association Between Combined NLR/mGPS Score and Clinicopathological Factors

When patient demographic, clinical, and pathological factors were considered, there were no statistically significant associations with the combined NLR/mGPS score (Table 3).

**Table 3:** Clinicopathological characteristics of esophagogastric cancer patients undergoing treatment with curative intent, grouped by combined neutrophil to lymphocyte ratio and modified Glasgow Prognostic Score

Characteristic		Combined NLR/mGPS			*p* Value
		NLR < 3	NLR > 3 + mGPS = 0	NLR > 3 + mGPS > 0	
*N*		152 (64)	55 (23)	32 (13)	–
*Patient characteristics*					
Age, years	< 65	74 (49)	25 (46)	10 (31)	0.422
	65–75	61 (40)	26 (47)	20 (63)	
	> 75	17 (11)	4 (7)	2 (6)	
Sex	Male	106 (70)	38 (69)	25 (78)	0.447
	Female	46 (30)	17 (31)	7 (22)	
BMI, kg/m^2^	< 20	4 (3)	4 (8)	2 (7)	0.092
	20–24	52 (36)	15 (30)	13 (47)	
	25–29	57 (40)	19 (38)	11 (39)	
	> 29	31 (21)	12 (24)	2 (7)	
Smoking	Never	48 (32)	14 (27)	12 (43)	0.990
	Ex-smoker	69 (46)	24 (46)	9 (32)	
	Current	32 (22)	14 (27)	7 (25)	
ECOG	0	113 (76)	34 (63)	22 (76)	0.963
	1	27 (18)	18 (33)	7 (24)	
	2	9 (6)	2 (4)	0 (0)	
VO2 AT, mL/kg/min	< 11	41 (36)	16 (37)	11 (44)	0.468
	≥ 11	74 (64)	27 (63)	14 (56)	
Anemia	No	107 (70)	34 (62)	18 (65)	0.081
	Yes	45 (30)	21 (38)	14 (44)	
CT sarcopenia^a^	No	47 (35)	16 (35)	5 (19)	0.175
	Yes	86 (65)	30 (65)	21 (81)	
*Tumor characteristics*					
Tumor site	Esophageal	41 (27)	15 (28)	14 (44)	0.186
	Gastric	14 (9)	35 (65)	15 (47)	
	Junctional	96 (64)	4 (7)	3 (9)	
Histology	Adenocarcinoma	109 (92)	43 (96)	14 (82)	0.553
	SCC	10 (8)	2 (4)	3 (18)	
cTNM stage	1	19 (12)	2 (4)	2 (6)	0.295
	2	33 (22)	16 (29)	7 (22)	
	3	93 (62)	34 (63)	22 (69)	
	4A	6 (4)	2 (4)	1 (3)	
*Initial treatment outcome*					
Surgery—R0		94 (62)	33 (60)	14 (44)	0.031
Surgery—R1/R2		27 (18)	13 (24)	3 (9)	
Surgery—Inoperable		16 (10)	2 (4)	5 (16)	
Progression on NAC		10 (7)	4 (7)	6 (19)	
NAC complication/major toxicity		5 (3)	3 (5)	4 (12)	

Data are expressed as *n* (%)

*BMI* body mass index, *CT* computed tomography, *ECOG* Eastern Cooperative Oncology Group performance status, *VO2 AT* oxygen uptake at the aerobic threshold, *cTNM stage* clinical TNM stage, *mGPS* preoperative modified Glasgow Prognostic score, *NLR* neutrophil to lymphocyte ratio, *NAC* neoadjuvant chemotherapy, *SCC* squamous cell carcinoma, *SMI* skeletal muscle index

^a^CT sarcopenia was defined as SMI^b^ < 52.3 cm^2^/m^2^ if the BMI was < 30 kg/m^2^, or < 54.3 cm^2^/m^2^ if the BMI was ≥ 30 kg/m^2^ in men; and SMI < 38.6 cm^2^/m^2^ if the BMI was < 30 kg/m^2^, or < 46.6 cm^2^/m^2^ if the BMI was ≥ 30 kg/m^2^ in women

^b^SMI (cm^2^/m^2^) was defined as the skeletal muscle area (cm^2^) measured from the axial CT slice at the L3 vertebral level/height (m)^2^

### Association Between Combined NLR/mGPS Score and Initial Treatment Outcomes

The combined NLR/mGPS score was significantly associated (*p* = 0.031) with the initial treatment outcome (Table 3). As the degree of inflammation increased (from NLR ≤ 3 to NLR > 3 + mGPS = 0, then NLR > 3 + mGPS > 0), there was a significantly higher rate of progression on NAC (7% vs. 7% vs. 19%), significantly higher rate of medical complication or major toxicity preventing patients reaching surgery (3% vs. 5% vs. 12%), and lower proportion of eventual R0 resections (62% vs. 60% vs. 44%).

### Association Between Combined NLR/mGPS Score and Overall Survival

At univariable Cox regression (Table 4), ECOG (*p* = 0.001), combined NLR/mGPS score (*p* = 0.006), clinical TNM stage (*p* < 0.001), and R0 resection (*p* < 0.001) were significantly associated with OS. At multivariable Cox regression, the combined NLR/mGPS score remained independently prognostic (hazard ratio [HR] 1.28, 95% CI 1.02–1.61; *p* = 0.032), along with ECOG (HR 1.61, 95% CI 1.21–2.13; *p* = 0.001), cTNM (HR 1.34, 95% CI 1.04–1.73; *p* = 0.025), and R0 resection (HR 0.24, 95% CI 0.18–0.33; *p* < 0.001).

**Table 4:** Cox regression of factors associated with overall survival in esophagogastric cancer patients prior to neoadjuvant chemotherapy with initial curative intent

Variables	Univariable HR (95% CI)	*p* Value	Multivariable HR (95% CI)	*p* Value
Age	1.11 (0.89–1.39)	0.359	–	–
ECOG	1.51 (1.19–1.93)	0.001	1.61 (1.21–2.13)	0.001
Smoking	1.02 (0.84–1.25)	0.814	–	–
BMI, kg/m^2^	0.88 (0.74–1.04)	0.135	–	–
VO_2_ AT < 11, mL/kg/min	1.34 (0.97–1.85)	0.073	–	–
Anemia	1.28 (0.94–1.74)	0.119	–	–
CT sarcopenia^a^	1.02 (0.74–1.39)	0.922		
Combined NLR/mGPS	1.45 (1.12–1.88)	0.006	1.28 (1.02–1.61)	0.032
cTNM stage	1.53 (1.22–1.91)	< 0.001	1.34 (1.04–1.73)	0.025
R0 resection	0.24 (0.18–0.33)	< 0.001	0.24 (0.18–0.33)	< 0.001

*HR* hazard ratio, *CI* confidence interval, *ECOG* Eastern Cooperative Oncology Group performance status, *BMI* body mass index, *VO2 AT* oxygen uptake at the aerobic threshold, *CT* computed tomography, *mGPS* preoperative modified Glasgow Prognostic score, *NLR* neutrophil to lymphocyte ratio, *cTNM stage* clinical TNM stage, *SMI* skeletal muscle index

*CT sarcopenia was defined as SMI^b^ < 52.3 cm^2^/m^2^ if the BMI was < 30 kg/m^2^, or < 54.3 cm^2^/m^2^ if the BMI was ≥ 30 kg/m^2^ in men; and SMI < 38.6 cm^2^/m^2^ if the BMI was < 30 kg/m^2^, or < 46.6 cm^2^/m^2^ if the BMI was ≥ 30 kg/m^2^ in women

^b^SMI (cm^2^/m^2^) was defined as the skeletal muscle area (cm^2^) measured from the axial CT slice at the L3 vertebral level/height (m)^2^

When all patients who began treatment with curative intent (planned NAC then surgery), with a calculable combined NLR/mGPS score, were considered, use of the combined NLR/mGPS score stratified 5-year OS in addition to clinical TNM stage and ECOG (Table 5). Five-year OS was stratified from 66% in those with cTNM stage 1–2 disease and the lowest combined NLR/mGPS score, to 22% in those with cTNM stage 3–4 disease and the highest combined NLR/mGPS score (*p* < 0.001). Five-year OS was stratified from 53% in those with an ECOG performance status score of 0 and the lowest combined NLR/mGPS score, to 14% in those with an ECOG performance status score of 1–2 and the highest combined NLR/mGPS score (*p* < 0.001).

**Table 5:** Overall survival following initiation of treatment with curative intent based on pretreatment clinical TNM stage, ECOG performance status, and combined NLR/mGPS

	ECOG performance status				*p* Value
	0		1–2		
	*n*	5-year OS, % (SE)	*n*	5-year OS, % (SE)	
*Combined NLR/mGPS*					
NLR < 3	110	53 (5)	34	35 (8)	
NLR > 3 + mGPS = 0	34	35 (8)	20	35 (11)	
NLR > 3 + mGPS > 0	21	38 (11)	7	14 (13)	
					< 0.001

	Clinical TNM stage				
	1–2		3–4A		
	*n*	5-year OS, % (SE)	*n*	5-year OS, % (SE)	
*Combined NLR/mGPS*					
NLR < 3	51	66 (7)	95	37 (5)	
NLR > 3 + mGPS = 0	18	56 (12)	36	25 (7)	
NLR > 3 + mGPS > 0	8	38 (17)	23	22 (9)	
					< 0.001

*ECOG* Eastern Cooperative Oncology Group performance status, *mGPS* preoperative modified Glasgow Prognostic score, *NLR* neutrophil to lymphocyte ratio, *OS* overall survival, *SE* standard error

When those patients from this group who went on to R0 resection were considered (Table 6), the use of the combined NLR/mGPS score stratified 5-year OS in addition to pathological TNM stage. Five-year OS was stratified from 77% in those with pTNM stage 1–2 disease and the lowest combined NLR/mGPS score, to 0% in those with pTNM stage 3–4 disease and the highest combined NLR/mGPS score (*p* < 0.001).

**Table 6:** Overall survival following R0 resection based on pathological TNM stage and combined NLR/mGPS

	Pathological TNM stage				*p* Value
	0–2		3–4A		
	*n*	5-year OS, % (SE)	*n*	5-year OS, % (SE)	
*Combined NLR/mGPS*					
NLR < 3	69	77 (5)	16	41 (13)	
NLR > 3 + mGPS = 0	23	64 (10)	9	22 (14)	
NLR > 3 + mGPS > 0	7	100 (0)	4	0 (0)	
					< 0.001

*mGPS* preoperative modified Glasgow Prognostic score, *NLR* neutrophil to lymphocyte ratio, *OS* overall survival, *SE* standard error

Finally, when the small number of patients from this group who did not go on to R0 resection were considered, the combined NLR/mGPS score did not provide additional prognostic stratification in addition to ECOG performance status (*p* = 0.209).

## Discussion

The present study reports that a combination of the systemic inflammatory scoring systems NLR and mGPS is associated with OS when measured prior to commencing NAC with curative intent in patients with ECs. It was also significantly associated with initial treatment outcomes, including the proportion of patients with disease progression during NAC, with major toxicity or medical complications during NAC, and the proportion of patients eventually undergoing a successful resection. Furthermore, the addition of the combined NLR/mGPS score to the ECOG performance status, as well as early clinical stage disease, effectively stratified 5-year OS in this group of patients. The use of such a readily clinically available measure of the SIR along with clinical staging and performance status may be helpful in identifying those patients likely to have a very poor prognosis despite treatment with curative intent, and therefore in whom symptomatic management may be more appropriate. Conversely, this method of staging both the tumor and the host may help to identify those most likely to benefit from aggressive treatment.

Both mGPS and NLR have been widely studied in a variety of solid tumors, each being shown to provide prognostication independent of disease stage.^13^ Indeed, the presence of systemic inflammation, by an increasing number of scores and ratios, is almost universally associated with poor prognosis, including in EC.^17^ It is hypothesized that the inflammatory response is driven by host–tumor interactions,^18^ and that along with potentiation of a prometastatic environment,^19^ a relative suppression of the host adaptive immune system leads to disease recurrence and death in these patients.^20^ The mGPS combines the acute-phase proteins CRP and albumin, and therefore can be seen as a clinically readily available marker of cytokine production and protein metabolism during an SIR.^21^ In contrast, the NLR provides a gross picture of the cellular response to the inflammatory stimulus.^22^ As such, previous attempts have been made to combine the two systems, perhaps providing an even greater overview of the inflammatory response, with success at prognostication in colorectal cancer.^14^ Indeed, as far as the authors are aware, at present this is the first paper that externally validates the method of combining the NLR and the mGPS. It is therefore also the first to apply it to a different cancer type.

Although the measurement of systemic inflammation has until now provided prognostic information in solid tumors, its clinical use has been less clear. However, recently, the combination of mGPS and TNM staging has been shown to effectively stratify survival in colorectal cancer patients,^23^ while the combination of mGPS and ECOG performance status has been used to stratify both survival and symptom reporting in patients with palliative disease.^24^ The routine incorporation of readily available measures of systemic inflammation in the clinical setting might therefore be used during the staging process to allow for both staging of the tumor and the host.^25^ This might allow multidisciplinary teams to offer an optimal treatment plan, preventing high morbidity aggressive treatment for those patients deemed to have a very poor prognosis when host and tumor factors are considered. Furthermore, as the possibility of treatments directed at the host inflammatory response to cancer becomes clearer, it may be that such a method identifies patients for whom additional treatment options to negate the impact of the innate inflammatory response are available.^26^,^27^ Examples of this in the preoperative setting might include non-specific anti-inflammatory treatments or more specific immunotherapies, although current evidence in the neoadjuvant setting does not extend to ECs and is focused primarily on checkpoint inhibitors such as nivolumab.^28^,^29^

The main limitations of the present study were that it was carried out in a single center and with a relatively small sample size. Further validation from future multicenter studies in a larger independent patient cohort is needed. The inclusion of esophageal, junctional, and gastric cancers, as well as a small number of squamous carcinomas, may have influenced the survival analysis. Other potentially curative treatment options, including upfront surgery and radical chemoradiation, were not included to reduce heterogeneity, and therefore make it easier to draw conclusions from the results generated from a complex patient care pathway. However, given the present results, the examination of combination systemic inflammation scoring as part of EC staging in these other modalities is warranted. Furthermore, not all patients had preoperative CRP, albumin, and FBC measured, further reducing the sample size. Finally, certain pathological and postoperative variables, e.g. ypTNM stage, LVI, postoperative morbidity, etc., which are well-recognized prognostic factors, were not included in the multivariable model. This was deliberate as the focus of this work was the addition of the host SIR to prognostic factors determined during staging and prior to NAC, which might influence initial or ongoing management.

## Conclusion

The present study reports that a pretreatment systemic inflammation score using a combination of NLR and mGPS is associated with poorer survival in patients undergoing treatment with curative intent for ECs. The effective stratification of 5-year OS when used along with ECOG performance status and clinical TNM stage reinforces the importance of staging both the tumor and the host. If validated in prospective studies, such a combined staging method may prove useful when making multidisciplinary treatment decisions about whether to submit patients to aggressive treatment or consider symptomatic management. This is of special importance in this patient group due to the relatively high morbidity associated with NAC and esophagogastric resections, and the poor long-term outcomes in those patients with higher clinical stage, poor performance status, and the presence of significant systemic inflammation.

## Electronic supplementary material

Below is the link to the electronic supplementary material.

Supplementary material 1 **SUPPLEMENTARY Fig.** **1** Kaplan–Meier curves with log-rank analysis of overall survival in all patients with esophagogastric cancer treated with curative intent with planned neoadjuvant chemotherapy comparing (**a**) by clinical TNM stage (*p* < 0.001), (**b**) initial treatment outcome (*p* = 0.001), and (**c**) pretreatment combined NLR/mGPS (*p* = 0.005). *mGPS* modified Glasgow Prognostic Score, *NLR* neutrophil to lymphocyte ratio, *Cum* cumulative (JPEG 75 kb)
