# Supplementary figures and images for: Staging the Tumor and Staging the Host: Pretreatment Combined Neutrophil Lymphocyte Ratio and Modified Glasgow Prognostic Score Is Associated with Overall Survival in Patients with Esophagogastric Cancers Undergoing Treatment with Curative Intent
Source: Ann Surg Oncol. 2020 Sep 5;28(2):722–31. doi: 10.1245/s10434-020-09074-5 (PMC7801291; doi:10.1245/s10434-020-09074-5)

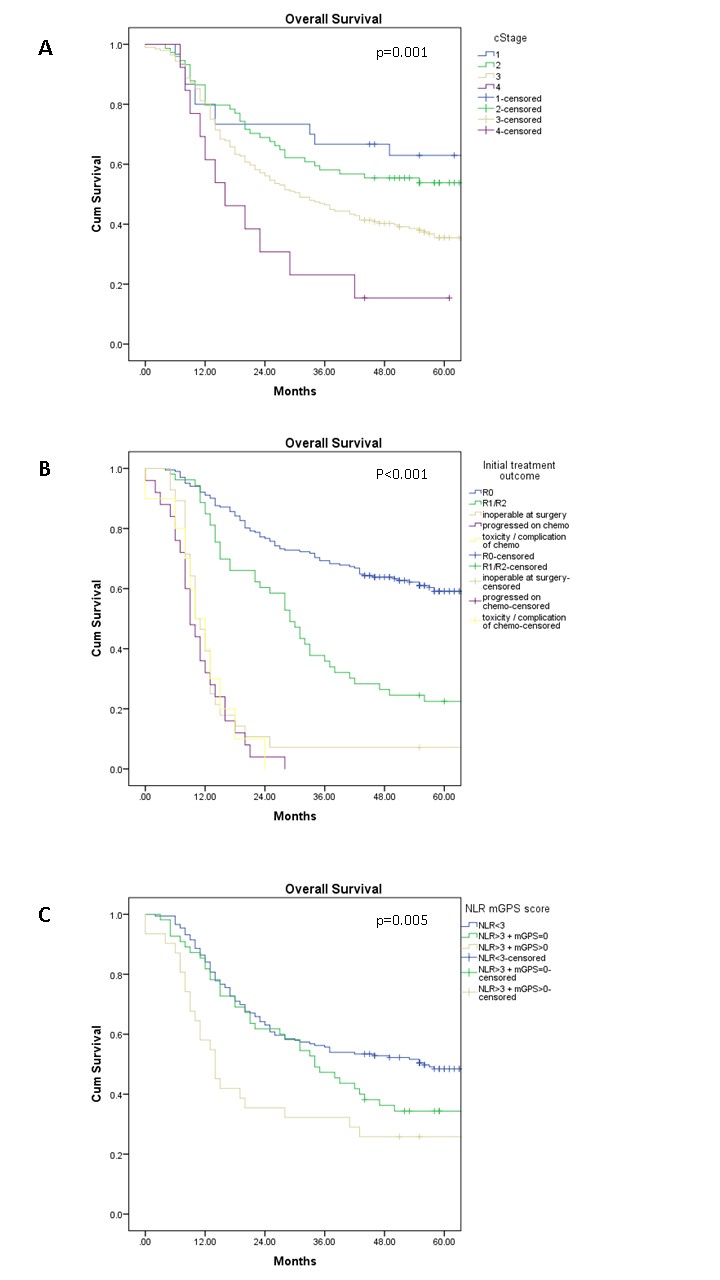

Supplement: Supplementary file 1 — Supplementary material 1 SUPPLEMENTARY Fig. 1 Kaplan–Meier curves with log-rank analysis of overall survival in all patients with esophagogastric cancer treated with curative intent with planned neoadjuvant chemotherapy comparing (a) by clinical TNM stage (p < 0.001), (b) initial treatment outcome (p = 0.001), and (c) pretreatment combined NLR/mGPS (p = 0.005). mGPS modified Glasgow Prognostic Score, NLR neutrophil to lymphocyte ratio, Cum cumulative (JPEG 75 kb) [file 10434_2020_9074_MOESM1_ESM.jpg]
